# Mathematical Models for Blood Flow Quantification in Dialysis Access Using Angiography: A Comparative Study

**DOI:** 10.3390/diagnostics11101771

**Published:** 2021-09-26

**Authors:** Nischal Koirala, Gordon McLennan

**Affiliations:** 1 Department of Chemical and Biomedical Engineering, Cleveland State University, Cleveland, OH 44115, USA; n.koirala@vikes.csuohio.edu; 2 Department of Biomedical Engineering, Lerner Research Institute, Cleveland Clinic, Cleveland, OH 44195, USA

**Keywords:** blood flow, bolus tracking algorithm, dialysis access, digital subtraction angiography, fluoroscopy, curve fitting, radiation dose

## Abstract

Blood flow rate in dialysis (vascular) access is the key parameter to examine patency and to evaluate the outcomes of various endovascular interve7ntions. While angiography is extensively used for dialysis access–salvage procedures, to date, there is no image-based blood flow measurement application commercially available in the angiography suite. We aim to calculate the blood flow rate in the dialysis access based on cine-angiographic and fluoroscopic image sequences. In this study, we discuss image-based methods to quantify access blood flow in a flow phantom model. Digital subtraction angiography (DSA) and fluoroscopy were used to acquire images at various sampling rates (DSA—3 and 6 frames/s, fluoroscopy—4 and 10 pulses/s). Flow rates were computed based on two bolus tracking algorithms, peak-to-peak and cross-correlation, and modeled with three curve-fitting functions, gamma variate, lagged normal, and polynomial, to correct errors with transit time measurement. Dye propagation distance and the cross-sectional area were calculated by analyzing the contrast enhancement in the vessel. The calculated flow rates were correlated versus an in-line flow sensor measurement. The cross-correlation algorithm with gamma-variate curve fitting had the best accuracy and least variability in both imaging modes. The absolute percent error (mean ± SEM) of flow quantification in the DSA mode at 6 frames/s was 21.4 ± 1.9%, and in the fluoroscopic mode at 10 pulses/s was 37.4 ± 3.6%. The radiation dose varied linearly with the sampling rate in both imaging modes and was substantially low to invoke any tissue reactions or stochastic effects. The cross-correlation algorithm and gamma-variate curve fitting for DSA acquisition at 6 frames/s had the best correlation with the flow sensor measurements. These findings will be helpful to develop a software-based vascular access flow measurement tool for the angiography suite and to optimize the imaging protocol amenable for computational flow applications.

## 1. Introduction

The ability to measure blood flow using digital images is of significant clinical interest that permits management, diagnosis, and treatment planning of vascular disease [1,2,3]. Autogenous fistula and prosthetic grafts are the most common vascular access types in end-stage renal disease (ESRD) patients that provide “access” to systemic circulation for high-efficiency dialysis [4,5,6]. Because of artificial shunting (fistula/graft), the fistula vein registers an abnormal surge in blood flow and invokes a complex pathophysiological process to accommodate the neo-fluidic stress [7,8]. This results in vascular remodeling (intimal hyperplasia) that, over time, leads to narrowing of the lumen and predisposes to stenosis and thrombosis [9,10,11,12]. Corrective angioplasty (percutaneous transluminal angioplasty) restores the hemodynamic function [13]. As per the National Kidney Foundation Kidney Disease Outcomes Quality Initiative (NKF/KDOQI), the minimum flow threshold to ensure patent access is 400–500 mL/min for arteriovenous fistula and 600 mL/min for arteriovenous grafts [14]. Based on these guidelines, the measurement of post-procedural blood flow can provide a reliable assessment of the radio-surgical endpoint.

The estimation of dialysis access blood flow is currently possible in all imaging modalities, including magnetic resonance (MR) [15,16], computed tomography (CT) [17], or ultrasound imaging [18,19]. The high costs of MR and CT scanning and the related logistic factors prevent its routine use for clinical examination and insurance reimbursement purposes. While the ultrasound technology is relatively inexpensive and does not involve ionizing radiation, multiple factors contribute to the poor estimation accuracy with ultrasound imaging. The error stems from the inaccuracy of the Doppler angle measurement of beam alignment with the vessel’s interface [20,21]. A deviation of > 20° in Doppler angle measurement leads to over 50% error in flow estimation [18]. The poor signal-to-noise ratio limits the ability to accurately measure the cross-section—a factor critical for flow estimation [22]. The attenuation difference between the blood and the tissue is also a concern that causes non-uniform insonation and scattering of the beam frequency [23,24]. Further, the accuracy and interpretation of the ultrasound data depend on the probe type, operator’s skill set, and experience, making the examination subjective [25,26]. Conversely, C-arm fluoroscopy is extensively used for intra-procedural guidance and imaging during dialysis access intervention and has the potential to quantify flow. However, to date, commercial application to measure access flow using fistulagrams is not yet available. The convenience of having an integrated flow measurement application in the angiography suite enables a cost-effective solution and provides an objective assessment of various endovascular procedures.

There have been various efforts to model the circulatory system to study the vascular mechanics and pathophysiological conditions in cardiovascular disease [27,28,29,30]. Polanczyk and colleagues introduced a novel artificial circulatory model (ACM) to simulate hemodynamics under various physiological flow conditions and measured the mechanical response of the vascular tissues [29]. The ACM- an ex vivo computer-controlled bioengineered reactor, was designed to mimic the human circulatory system to study vessel’s wall compliance in real-time. To effect, an “artificial heart” was presented to independently vary the ejection pressure, ejection volume, and frequency of pulsation, and a novel vision acquisition system was conceived to record the 3D-geometrical changes of the vessel in response to different flow stimuli. Vascular biomechanics were simulated under various physiological flow conditions and the corresponding wall deformation responses were validated with the medical data reconstructed with 2D-speckle tracking technique that showed good agreement [29]. In another study from the same investigator, a novel vision-based system was proposed to spatially assess abdominal aortic aneurysm and its wall deformation in a water environment [30]. The non-invasive vision-based system (NIVBS) consisted of a set of nine cameras oriented in circular geometry and custom (self-made) computer vision algorithms were applied to interrogate aneurysm sac wall deformation. Aortic biomechanics were reconstructed in a water environment to simulate physiological pressures from surrounding organs, and the aneurysm diameter and wall deformation were calculated. These experimental data were correlated with AngioCT and 2D speckle–tracking echocardiography that showed good correlation [30]. Xaplanteris et al. have proposed a continuous thermodilution-based approach to calculate absolute coronary blood flow (*Q*) and microvascular resistance (*R*) in human subjects [31]. The authors used a monorail thermodilution catheter equipped with temperature and pressure sensors to measure the flow and resistance by injecting a saline bolus at room temperature. The quantification of microcirculation and resistance allows a simple and operator-independent assessment and treatment of coronary microcirculatory dysfunction in patients with angina. The absolute coronary blood flow and resistance were calculated based on the following equation [31]:

(1) $$Q=c_{p}\left[\frac{\left(T_{b}-T_{i}\right)}{\left(T_{b}-T\right)}-1\right]Q_{i}+Q_{i}$$

where, *Q_i_* is the saline infusion rate (mL/min), *T_b_* is the temperature of blood before infusion (≈37 °C), *T_i_* is the temperature after infusion at catheter exit, *T* is the homogeneous temperature of the blood-saline mixture, and *c_p_* is a constant related to the thermal properties of blood and saline (=1.08). Upon simplification and eliminating negligible quantities, *Q* can be represented as [31]:

(2) $$Q=1.08\frac{T_{i}}{T}Q_{i}$$

*R* was calculated following Ohm’s law analogy:

(3) $$R=\frac{P_{d}}{Q}$$

where, *P_d_* is the pressure at the distal coronary.

Medical image analysis allows extraction of the data that are normally buried in the images and application of relevant mathematical models to extract the physiological parameters of interest. These hemodynamic metrics, such as velocity, pressure/stress, wall diameters, etc., can be correlated to the disease pathology and can foster our understanding of disease initiation/progression and seek rational solutions. Khanmohammadi and colleagues have proposed a multi-step automatic method to estimate blood flow velocity in coronary arteries based on cine X-ray angiographic sequences [32]. X-ray coronary angiography is an invasive routine procedure performed to assess coronary artery disease in patients with angina pectoris. The study recruited 21 patients who did not have obstructive coronary artery disease and coronary flow velocity was measured. The process of velocity calculation involved segmentation of coronary arteries and area calculation, removal of heart motion, polynomial fitting (7th order) on area sequences for start/stop time determination of indicator passage, and velocity calculation based on the principles of indicator propagation. The calculated flow velocity significantly correlated with the transthoracic Doppler recordings [32]. Similarly, intracoronary frequency-domain optical coherence tomography (FD-OCT) was used to examine blood flow rate and velocity in coronary artery stenosis [33]. The measurements were correlated with fractional flow reserve (FFR), which is considered a gold standard for the evaluation of coronary stenosis. OCT uses near-infrared light as opposed to sound waves by intravascular ultrasound (IVUS) and provides high-resolution imaging for lumen measurement. The study evaluated 20 coronary stenoses in 15 patients successively with quantitative coronary angiography (QCA), FFR, and FD-OCT. The authors reported a statistically significant correlation between FD-OCT and FFR calculated flow velocity that suggested the potential value of FD-OCT for the assessment of coronary artery disease against QCA and IVUS imaging modalities [33]. In our study, we are interested to quantify blood flow in dialysis access based on the tools available in the radiological suite. Without additional logistic requirements, the software-based approach, when fully validated, is envisioned to seamlessly integrate into the existing image analysis suite as an installable add-on package.

The purpose of this study is to evaluate image-based flow quantification methods for dialysis access using bolus tracking algorithms and curve-fitting models and to inform the most pertinent method applicable for flow quantification. In our previous study, we quantified blood flow based on the images of digital subtraction angiography (DSA) acquisition and did not employ image pre-processing or curve-fitting models that resulted in poor reproducibility and inaccuracy of measurement [34]. Here, it is hypothesized that the use of curve fitting reduces variabilities in time measurement and results in an enhancement of accuracy and consistency of measurement.

## 2. Materials and Methods

A radiographic technique to measure blood flow in artificial dialysis access is presented here. Blood flow measurement using angiography is based on tracking the passage of the radiopaque (iodinated) contrast material (CM) at two regions of interest (ROIs) and measuring the corresponding transit time, spatial displacement, and vessel dimension [35]. Using a flow phantom model and a C-arm fluoroscope, hemodynamics from the injection of CM was studied.

### 2.1. Transit Time Algorithm

The transit time algorithm determines the time taken by the contrast bolus to travel between two user-specified ROIs. A time-dependent intensity map of the transiting bolus is generated at each ROI, also known as time-density curves (TDC), provides the basis for time calculation. Based on various calculation methods (algorithms), the characteristic feature(s) of the first curve is tracked and compared with the second curve [36,37]. Herein, we discuss two major bolus tracking algorithms for transit time estimation. A comprehensive review of other applicable transit time algorithms has been discussed elsewhere [38,39,40].

#### 2.1.1. Peak-to-Peak (PP) Algorithm

The PP algorithm calculates the bolus arrival time based on the time corresponding to the appearance of peak (maximum) intensity at each ROI. The transit time (*τ_PP_*) is then calculated as the difference between the bolus arrival times at each ROI [35].

[*t_ROIx_* (*x* = 1, 2)]*_PP_* = *t*(*P_ROIx_*)*_max_* (4)

(5) $$\tau_{PP}=\Delta t={\left[t_{ROI2}\right]}_{PP}-{\left[t_{ROI1}\right]}_{PP}$$

where, *t_ROIx_* is the time corresponding to the appearance of maximum peak intensity (*P*)_max_ at *ROI_x_*, and Δ*t* (=*τ_PP_*) is the required bolus transit time.

#### 2.1.2. Cross-Correlation (CC) Algorithm

The CC algorithm calculates the transit time based on a process known as template matching, i.e., the TDC obtained at one ROI is compared with the TDC at another ROI [34,41]. The process of CC requires time-shifting of one curve against the other until a maximum overlap or an optimal match is obtained. The time for which the CC function (*φ*) is maximized is the required transit time or the initial delay between the two curves.

(6) $$\varphi_{P1P2}\left(\tau \right)=\frac{1}{N}\overset{N}{\underset{t=1}{\sum }}P_{1}\left(t\right)P_{2}\left(t-\tau \right)$$

where *P*_1_ and *P*_2_ are the time-density functions corresponding to *ROI1* and *ROI2*, respectively, *N* is the number of temporal data samples, and *τ* = *N* × Δ*t*; Δ*t* is the unit time interval (time resolution) of the imaging modality.

### 2.2. Curve-Fitting Models

The calculation of the transit time using the raw signal data is prone to errors from random fluctuations of intensity, quantum noise from image acquisition (image mottle) [42], and recirculation [43]. The curve-fitting function provides a mathematical description of the TDCs based on the statistical properties and assumptions of the underlying distribution. In theory, the curve-fitting function reconstructs a noise-free reference curve amenable for computational purposes by isolating the bolus first pass, which can be used for the estimation of transit time. In this study, we evaluated three curve-fitting models.

#### 2.2.1. Gamma-Variate (GV)

The GV function is one of the most commonly used curve-fitting methods to model the indicator-dilution phenomena in hemodynamic studies [15,19,44], first described by Thomson et al. [45]. It replaces the original intensity curve with a characteristic curve that has a sharp ascending slope, a slowly decaying tail, and a compact spread (narrow curve width) that closely resembles a right-skewed Gaussian curve. The fitting parameters can be optimized to ensure the best fit to represent a particular phenomenon.

Mathematically, the GV function can be written as [45]:

(7) $$C\left(t\right)=K_{a}{\left(t-AT\right)}^{\alpha }e^{-\left(t-AT\right)/\beta }$$

where, *C*(*t*) is the indicator concentration at time, *t*, *K_a_* is the constant scale factor that determines the level (maximum magnitude) of *C*(*t*), *AT* is the contrast appearance time, and *α* and *β* are arbitrary parameters that determine shape (rate of decay) of the curve.

#### 2.2.2. Lagged Normal

The lagged normal distribution was first described by Bassingthwaighte et al. to model the dispersion of an indicator in the flowing fluid [46]. It was derived to overcome Poiseuille’s flow model (parabolic flow) for estimating blood flow in the vasculature, which is often skewed and turbulent. It was shown that the model can fit a wide variety of dilution curves. Lagged normal function describes the indicator-dilution phenomenon based on the compartmental model of tissue perfusion that combines the normal distribution (Gaussian function) with an exponential function to represent the trailing edge [47]. In particular, the Gaussian function provides the basis for random dispersion with turbulence, and the exponential function models the mixing that occurs in the chambers [48]. Lagged normal distribution was used to model the blood flow from a large vessel into a microvascular bed [19].

In the simplest form, we can represent lagged normal distribution by the convolution of two functions: a Gaussian function and an exponential function [19,46,48].

(8) $$C\left(t\right)=\left[f*g\right]\left(t\right)=\int_{-\infty }^{\infty }f\left(\tau \right)g\left(t-\tau \right)d\tau =\int_{-\infty }^{t}f\left(\tau \right)g\left(t-\tau \right)d\tau$$

(Since, *g* (*t − τ*) = *0* for *t < τ*, the upper limit of the integral is *t*)

Where

(9) $$f\left(t\right)=\frac{1}{\sqrt{2\pi \sigma^{2}}}e^{-{\left(t-\mu \right)}^{2}/\left(2\sigma^{2}\right)},-\infty <t<\infty$$

(10) $$g\left(t\right)=\lambda e^{-\lambda t},\;t\geq 0$$

where *µ* and *σ*^2^ represent the mean transit time and variance of the Gaussian function. The parameter *λ* is the rate constant of the exponential function.

Solving for *C*(*t*) resolves into:

(11) $$C\left(t\right)=\frac{A}{2}K\left[1+\mathrm{erf}\left(L\right)\right]$$

where *A* is the area under the curve, and *erf*(.) indicates the error function, defined as:

(12) $$erf\left(x\right)=\frac{2}{\sqrt{\pi }}\int_{0}^{x}e^{-t^{2}}dt$$

The parameters *K* and *L* are given as:

(13) $$K=\lambda e^{\left(-\lambda t+\lambda \mu +\frac{1}{2}\lambda^{2}\sigma^{2}\right)}$$

(14) $$L=\frac{t-\mu -\lambda \sigma^{2}}{\sqrt{\left(2\sigma^{2}\right)}}$$

#### 2.2.3. Polynomial (6th Degree)

The lower degree polynomial fails to fit the raw data adequately to resemble a typical TDC. Conversely, higher-order polynomials fit the data quite well along with noise, which is undesirable. Therefore, the process of fitting and selection (optimization) of polynomial order requires a cautious approach to balance the accuracy of fit against noise inclusion.

The sixth-order polynomial, used in this study, can be written as:

(15) $$C\left(t\right)=a_{1}+a_{2}t+a_{3}t^{2}+a_{4}t^{3}+a_{5}t^{4}+a_{6}t^{5}+a_{7}t^{6}$$

(16) $$C\left(t\right)=\overset{7}{\underset{i=1}{\sum }}a_{i}t^{i-1}$$

where *C*(*t*) is the contrast concentration at any time, *t*, and *a_i_* is the fitting constant to be determined.

### 2.3. Optimization of Curve-Fitting Parameters

**Figure 1:**
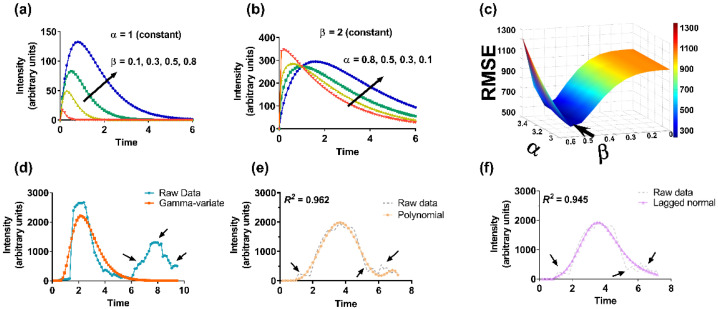
Illustration of curve fitting and optimization process. (**a**–**d**) GV curve fitting by optimizing unknown parameters, (**a**) *α* and (**b**) *β*. Change in *β* changes the magnitude whereas variation in *α* changes the shape of the curve (rate of decay), (**c**) selection of *α* and *β* based on least root mean square error (*RMSE*) (arrow), (**d**) raw data, and final reference curve based on *GV* function. Reference curves based on (**e**) polynomial, and (**f**) lagged normal curve-fit functions. Recirculation and random intensity variations (shown by arrows in raw data) are absent in the fitted curves.

Initially, an approximated (coarse) value of the unknown parameters was obtained by plugging random numbers. Based on the direction of the reduction (minimization) of the cost function (root mean square error, *RMSE*), the parameters were further optimized in fine increments. As an example, we have provided the process of optimization for the GV curve-fit function (Figure 1). The results of other curve-fitting models are depicted. (Note: the polynomial fitting was executed using MATLAB’s inbuilt “*polyfit*” function).

### 2.4. Distance and Cross-Sectional Area Calculation

**Figure 2:**
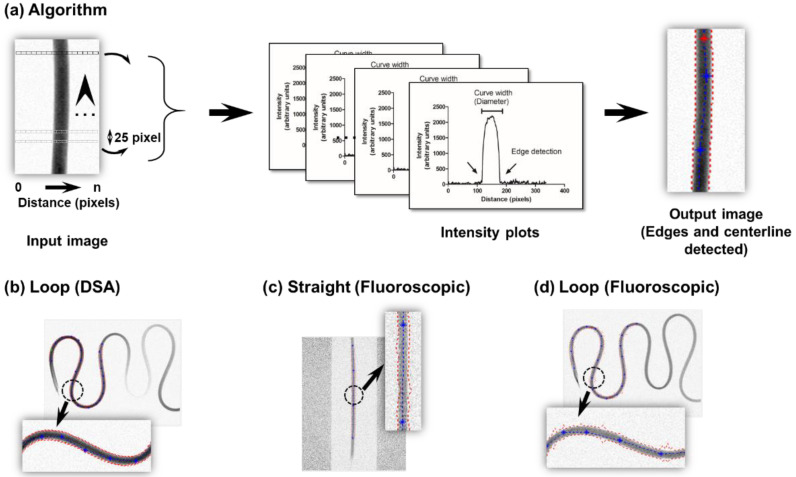
Edge detection method for area and distance calculation. (**a**) Algorithm elucidating the process of edge detection for an input image based on multiple distance-intensity plots obtained at discrete intervals (25 pixels) between the ROIs (up arrowhead indicates direction of fluid flow; dye propagation distance and edge detection are evaluated along this direction), (**b**) extracted edges, and the centerline based on a loop configuration for DSA acquisition. For fluoroscopic imaging, the extracted edges and centerline are based on a (**c**) straight tube, or (**d**) loop configuration. Dotted red lines indicate edges, and dotted blue lines indicate centerline (zoomed image in inset).

The edge detection method was specifically developed to calculate the spatial distance traveled by the contrast bolus and the vessel’s cross-sectional area [34,35]. Because the injection of the CM enhances the vessel (contrast opacification), edges can be determined by discriminating the intensity interior of the vessel with the image background. The algorithm for edge detection is illustrated (Figure 2). For every row, assume each box in the image to be of unit pixel length of given intensity (white pixel- low intensity, black pixel- high intensity, gray pixel- in between intensity (sense of pixel intensity was inverted to guide the eye)). The assignment of a row begins with the coordinates of *ROI1* and ends with the coordinates of *ROI2*. Each row within the ROI produces exactly one distance-intensity plot. In this manner, multiple distance-intensity curves were plotted along the vessel’s length (bolus travel path) at discrete intervals (25-pixel separation). At the vessel interface (either edge), there was a sharp change in the contrast bolus intensity. The initial cut-off intensity to declare edge transition was set at 5% of the maximum opacification and fine-tuned until an accurate edge identification could be reliably made (settled at ~12%). After the edges were detected, the centerline was calculated as the mean distance between the two extracted edges. The contrast traversal distance was calculated based on the pixel separation between the two ROIs along the extracted centerline. Because the image mottle was more prevalent on fluoroscopic images, the extracted edges and centerline were relatively noisier over DSA acquired images.

The cross-sectional area was calculated assuming a circular geometry. The access diameter was determined from the curve width of the distance-intensity plot, generated previously (edge detection). To accommodate for changing vessel’s diameter often encountered in clinics, the mean cross-sectional area approach was adopted for flow estimation, i.e., the average of multiple cross-sectional areas sampled at discrete intervals (25-pixel separation) between the ROIs was used.

### 2.5. Flow Phantom Set Up

**Figure 3:**
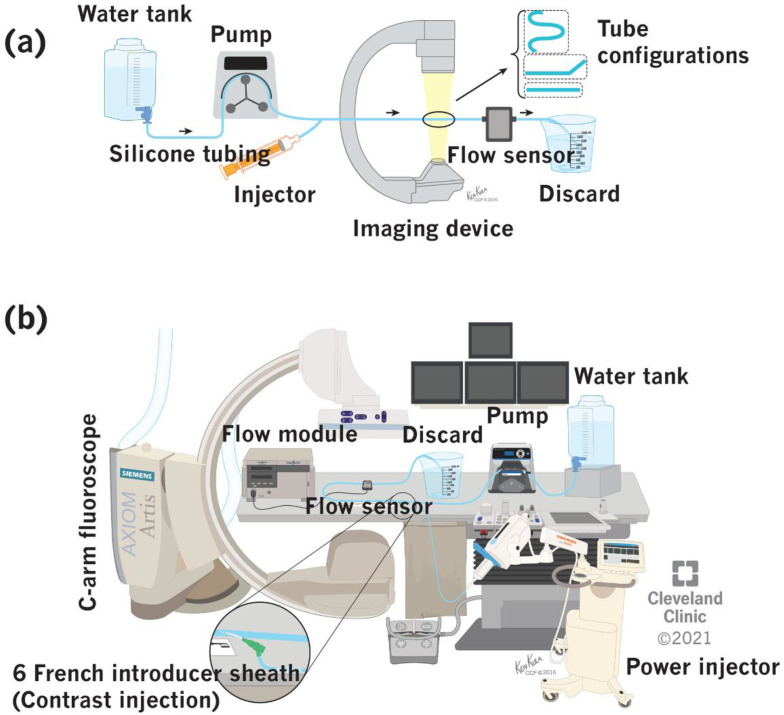
Benchtop set up for simulation of blood flow in a flow phantom model. (**a**) Block diagram (arrows indicate direction of flow), (**b**) experimental set up. (Reprinted with permission, Cleveland Clinic Center for Medical Art & Photography © 2021. All rights reserved).

A water-based benchtop model for simulating dialysis access was constructed (Figure 3). It comprised a water tank, a peristaltic pump (EW-07528-10 & EW-77200-50, Cole-Parmer Instrument Company, Vernon Hills, IL, USA), power injector (Mark V Provis, Medrad Inc., Warrendale, PA, USA), in-line flow sensor (ME6PXN, Transonic Systems Inc., Ithaca, NY, USA), and a discard serially connected in an open circuit configuration via a silicone tubing [34,49]. Additionally, three different tubing configurations (straight, angular, and loop) were tested. The entire setup was placed over an angiographic table and imaged in DSA and fluoroscopy filming modes using a C-arm fluoroscope.

The DICOM images obtained from the above imaging experiments were fed offline into an ordinary personal computer equipped with a GUI-based access flow computational model constructed entirely in MATLAB. It required the users to identify two ROIs in the bolus flight path and had the option to use various curve-fitting models and interpolation functions for flow calculation.

### 2.6. Image Acquisition Protocol

The imaging protocol was developed to resemble a realistic clinical imaging environment [34,49]. DSA images were acquired at 3, and 6 F/s; fluoroscopic images were acquired at 4, and 10 pulses/s (P/s). The total duration of imaging was set at 8 s, and CM was power injected via a sidearm 6 French introducer sheath. The contrast injection was synchronized with the imaging function in the C-arm fluoroscope and injected at 3.33 cc/s for 3 s (10 mL over 3 s) with an initial delay of 1 s. The imaging duration and initial injection delay were optimized based on computational experience that required a pre-contrast phase along with bolus wash-in and wash-out segments for enhanced accuracy.

## 3. Results

**Figure 4:**
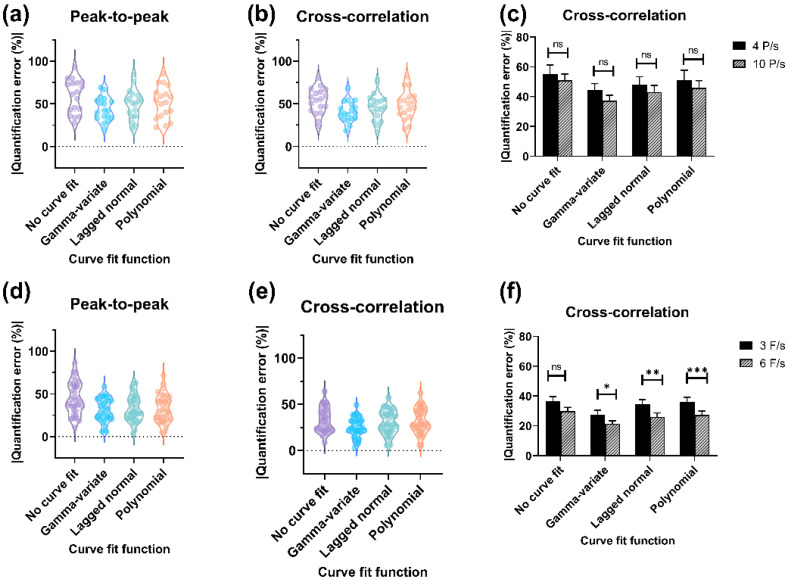
Evaluation of quantification error, *Upper panel:* Fluoroscopic imaging, *Lower panel:* DSA acquisition. Violin plots illustrating overall quantification error with PP and CC, algorithms for (**a**,**b**) fluoroscopic imaging, and (**d**,**e**) DSA acquisition. Bar graphs showing the absolute quantification error (mean ± SEM) with various curve-fitting models at different sampling rates for (**c**) fluoroscopic, and (**f**) DSA acquisition. The shape of the violin plot indicates overall sample distribution, and the central region specifies the median and interquartile range. ns—non-significant, *—0.044, **—0.013, ***—0.022.

**Figure 5:**
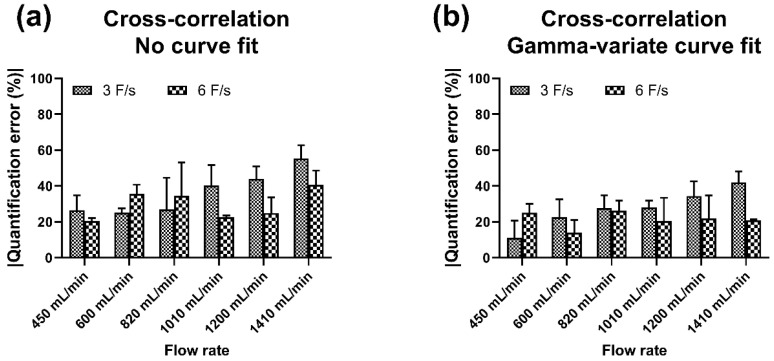
Evaluation of quantification accuracy of CC algorithm at different flow rates for DSA acquisition. Absolute quantification error (**a**) without curve fit, and (**b**) with GV curve fit.

The overall results obtained with each algorithm with and without different curve-fitting models were plotted to examine a general trend of accuracy and reproducibility for each imaging mode (Figure 4). We quantified the accuracy in terms of absolute percent quantification error, i.e., absolute percent deviation from in-line flow sensor measurement (ground truth) for each observation. For both the imaging modes, the CC algorithm had the highest accuracy (least quantification error) and least deviation (high precision). Among the different curve-fitting models, the GV curve fit had the best accuracy and precision of measurement. For DSA acquisition, the lowest quantification error (mean ± SEM) was obtained with CC + GV curve fit (6 F/s), and the highest quantification error of 52.9 ± 4.3% was obtained with the PP algorithm without curve fit (4 F/s). Since the CC algorithm had the best overall accuracy, the effect with/without curve fit on accuracy at different sampling rates was evaluated (Figure 4c). The quantification error with GV curve fit at 4 F/s was 27.6 ± 2.7% and at 6 F/s was 21.4 ± 1.9% (highest accuracy). Similarly, for fluoroscopic imaging, the least quantification error (mean ± SEM) was obtained with the CC algorithm + GV curve fit (10 P/s), and the highest quantification error of 64.1 ± 6.5% was obtained with PP algorithm without curve fit (4 P/s). The variation in quantification accuracy with the CC algorithm at different pulse rates as a function of curve fit or none (no curve fit) was plotted (Figure 4f). The quantification error with the GV curve fit at 4 P/s was 44.4 ± 4.3% and at 10 P/s was 37.4 ± 3.6% (highest accuracy). The quantification error of the PP algorithm with different curve-fitting models for both imaging modalities (DSA acquisition and fluoroscopy) is illustrated (Figure S1). The quantification error with the GV curve fit at 6 F/s was 26.3 ± 2.9% and at 10 P/s was 42.2 ± 4.3%. The variation in quantification accuracy with the CC algorithm as a function of flow rate for DSA acquisition is depicted (Figure 5). The results are shown for the CC algorithm with or without GV curve fit because of the consistency of measurement, as stated previously (for PP algorithm, refer to Figure S2). Data show DSA acquisition at 6 F/s with CC algorithm + GV curve fit had the least quantification error when the flow rates were varied. These results affirmed that the CC algorithm with GV curve fit had the highest accuracy and precision of measurement and is best suited for flow computational studies.

**Figure 6:**
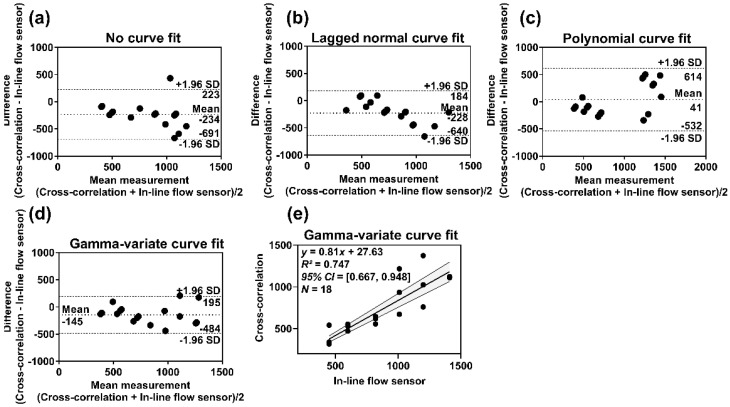
(**a**–**d**) Bland–Altman plots for DSA acquisition showing the bias (mean) and limits of agreement (mean ± 1.96 × SD) for CC algorithm with/without curve fitting versus in-line flow sensor measurement, (**e**) Scatter plot showing the correlation between measurements of CC algorithm with gamma-variate curve fit versus in-line flow sensor reading, along with 95% confidence bands of the best-fit line. (For fluoroscopic imaging, refer to Figure S3).

The bias, limits of agreement (LOA), and 95% confidence interval (CI) of the mean quantification error for the DSA acquisition and fluoroscopic imaging were assessed (Table 1). Bias and LOA were calculated based on Bland-Altman’s theory [50]. Bias indicates the systemic tendency of the method to under- or overestimate the actual measurement and calculated as the mean of the measured differences with in-line flow sensor measurement. LOA indicates agreement of the calculated value with the measured (actual) value and depicted by a bandwidth (bias ± 1.96 × SD of differences), where 95% of the differences are expected to lie (the narrower the bandwidth, the more precise is the measurement). Since the CC algorithm had the least quantification error, we have shown BA analyses for this method (DSA acquisition: Figure 6, Fluoroscopy: Figure S3). The CC algorithm with GV curve-fitting had the best agreement with the measured values. The 95% CI for the bias and LOA bandwidth was also the least indicating reproducibility (precision/ repeatability) of the measurements. The CC algorithm with GV fit at 6 F/s exhibited a good correlation with the actual flow (*r* = 0.898). We made similar observations for fluoroscopic imaging, except that the range was wider, which indicated large variabilities of measurements over DSA acquisition. The correlation (*r*) between measured flow rates obtained with CC algorithm + GV fit at 10 P/s and in-line flow sensor was 0.334.

Since the use of C-arm angiography involves ionizing radiation, the radiation burden at different sampling rates was evaluated for DSA and fluoroscopic examinations (Figure S4). In clinics, radiation doses are usually stated in terms of air kerma (AK) and air kerma area product (AKAP, or dose area product (DAP)) since these quantities are readily available in the modern fluoroscopic machines (angiographic workstation) and collected at the end of the procedure for record-keeping purpose. These parameters can be used to derive the peak skin dose (PSD) and effective dose (ED), which are more sensitive and specific quantities for measuring the effects of radiation, i.e., tissue reactions (deterministic effect (PSD)) and stochastic effects (radiation-induced cancer (ED)). It was found that the radiation dose from these procedures was substantially low to invoke any significant detrimental effects. Radiation dose varied linearly and increased proportionately with the sampling rate.

## 4. Discussion

This study aimed to identify key algorithm(s) and curve-fitting model(s) that can provide an accurate and consistent estimation of the access flow should a software application be developed. We tested two transit time algorithms and three curve-fitting models to find an optimum method for quantifying dialysis access flow. It was found that the CC algorithm with GV curve fit, compared to other methods, had the most accurate and consistent description of the access flow (DSA—6 F/s, Fluoroscopic—10 P/s). The CC method uses the area under curve approach to calculate the optimum time delay that makes it comparatively immune to random fluctuations of the intensities [34,41,49].

During the radiological intervention of the dialysis access, a concentrated bolus of CM is injected into the bloodstream that enhances the path of the bolus flow [51,52,53]. While the visibility of the vascular network is fundamental for clinical diagnosis, it also contains intrinsic hemodynamic information that can be exploited for measuring physiological flow [34,35]. The estimation of bolus arrival time using raw data is challenging, as it contains noisy signals and is devoid of any characteristic shape [49]. Noisy components appear on the dilution curves because of the non-homogenous composition of the blood-contrast mixture and technological factors associated with imaging [54]. Since bolus arrival time depends on the shape of the enhancement curve, fluctuations of intensities change the curve contour and exert a negative impact on computational accuracy. Therefore, a uniformly enhanced curve profile with minimum intensity fluctuations is expected for quantification, which can be accomplished through the use of curve fitting. The GV curve-fit function had the best results among all other curve-fitting methods. It provided a close similarity to the actual physiological flow with a relatively simpler optimization process. The lagged normal distribution could also model the dilution curves and get rid of unwanted random intensity outliers; however, the accuracy and repeatability were lower than that of the GV curve-fitting approach. Conversely, polynomial fitting did not accurately model the TDC and more likely followed the noise inherent in the dilution curves that resulted in poor computational accuracy.

During clinical imaging, the sampling rate and imaging duration are usually kept low to avoid a substantial amount of radiation dose to the patient and the clinical staffs in the operating room [55,56,57]. The increase in sampling rate affects the computational accuracy and the radiation dose as it relates to the number of samples available for computational purposes and the X-ray beam on time, respectively. At a low sampling rate, observations were obtained at infrequent intervals that amounted to a low number of data samples and poor depiction of the bolus passage. At a high sampling rate, large amounts of data (better image quality) were rapidly generated, albeit at the cost of increased radiation burden. Thus, a balance between accuracy and acceptable radiation dose needs to be ascertained for a clinically realistic environment. The radiation burden increased linearly with the increase of frame rate or pulse rate but was substantially low to invoke any radiation-induced injury. The sampling rate of 3 F/s (DSA) or 4 P/s (fluoroscopy) had the least accuracy. With the increase in sampling rate to 6 F/s or 10 P/s, computational accuracy was improved. The quantification accuracy and precision with the DSA acquisition were superior to that of the fluoroscopic imaging. The evaluation of fluoroscopic imaging was motivated by its extremely low radiation footprint. However, because of the use of a low radiation X-ray beam, the images were noisier that affected the extraction of the cross-section area and transit time. The accurate estimation of the cross-sectional area is of paramount significance as it impacts the flow calculation to the second power of the radius (∝ *R^2^*). Likewise, the conversion of flow rate units (mL/s to mL/min) involves a factor of 60 that rapidly overestimates the calculated flow rate, including with a small degree of transit time calculation inaccuracy. Therefore, for clinical purposes, DSA acquisition at 6 F/s may be preferred. Waechter et al. used 3D rotational angiography to model the flow and to determine hemodynamic parameters based on the spatial and temporal progression of the contrast concentration in a phantom model [3]. The error rate was 5–10% of actual flow, and the images were obtained at 30 F/s. Similarly, Pereira and colleagues used 3D rotational angiography to study intra-aneurysm hemodynamics induced by flow diverter stents that led to progressive thrombosis and vascular remodeling [58]. The study was conducted on 24 patients with variable flow rates. Images were acquired at 60 F/s, and the correlation of flow amplitude with the prediction of aneurysm thrombosis was 88% (sensitivity) and 73% (specificity). A cross-correlation algorithm was used for flow quantification by Seifalian et al. [59]. Images were acquired at 25 F/s; the error of quantification was 20–50% for flow velocity ranging from 147 to 733 mm/s. Against this background, this study obtained an accuracy of 21.4 ± 1.9% with DSA acquisition at a low sampling rate of 6 F/s, which is a realistic frame rate for clinical applications.

The measurement of access flow involves a complex interplay of multiple factors that includes optimizing the parameters of injection and imaging duration and managing the radiation dose in a manner that maximizes the diagnostic value with minimum risk for radiation-induced injury [43,54,60,61]. Since the determination of blood flow is entirely dependent on the analysis of radiological exams, it is necessary to have good quality images with adequate temporal information to enable accurate quantification [62,63]. With these facts in mind, we developed a custom injection and imaging protocol for use with this study. The objective of the customized protocol was to record the pattern of physiological flow during image acquisition, possibly with the lowest radiation burden. We emphasize the use of a power injector over the handheld injection method for administering CM as human factors such as the rate and volume of injection can affect flow dynamics. The use of a power injector produces highly uniform enhancement curves without operator dependency and delivers precision in time calculation. In addition, the concentration of the CM directly affects the quality of enhancement. In this study, we used CM at full strength (100%) and did not experiment with various dilutions since the volume of administration was considerably low to invoke any adverse reactions to CM non-allergic patients [64]. However, while the use of diluted or concentrated CM media may have no substantial impact on the analysis of DSA images, it can affect the analysis of fluoroscopic images, as the image quality is extensively degraded (poor signal-to-noise ratio).

We evaluated contrast enhancement curves to calculate the centerline and distance traveled between two regions of interest. We first applied this concept to a simple tube configuration followed by angled and loop configurations and could extract the centerline and distance traveled by the contrast bolus with reasonable accuracy.

This study has few limitations. First, we used water in lieu of blood and it may not necessarily correlate with the in vivo fluid dynamics. For instance, the hematocrit influence on blood flow was not studied. However, while the hematocrit does affect viscosity, the system was conceived as an internally controlled model with a rigorously validated gold standard (ultrasonic flow sensor) for the measurement of flow. Because we measure the same material with angiographic imaging and ultrasonic flow sensor, the composition of the flowing fluid may not exert a significant impact. Further hematocrit effects on flow mostly relate to capillary flow and the physiological effects of altering the cellular composition of the blood on the apparent viscosity of blood (Fahraeus effect) [65]. The fistula is a clinically constructed conduit in the peripheral circulatory system to facilitate dialysis with vessel’s dimension ranging up to 1–2 cm in diameter and registers a flow rate of 300–1000 mL/min (patent access). Due to the high bulk flow rate in the dialysis fistula, the homogenous blood-contrast mixture is rapidly dispersed in circulation [43]. For more realistic blood flow simulation, future studies need to examine blood analogs such as water-glycerol or xanthan gum-water-glycerol mixtures as potential test fluids [29,66]. Second, we chose a simple geometric construction of the vessel and applied circular cross-sectional model for area calculation. These assumptions were made to simplify the study and to avoid the use of 3D rotational angiography to acquire the third-dimensional parameter necessary for the reconstruction of a 3D model. The assumption of a circular cross-section made here may not necessarily hold for stenotic vessels, which are essentially malformed. Since the change in vessel geometry affects the flow regimen (oscillatory/turbulent flow), future study needs to investigate the use of a noncircular cross-sectional model, its impact on dispersive flow, and the technical aspects associated with image acquisition and analysis.

## 5. Conclusions

A radiological concept to estimate flow based on the principles of indicator propagation under the influence of bulk flow was presented. The measurement of blood flow provides the functional significance of stenosis and elucidates the severity of the pathology that permits immediate remedies [67,68,69]. The paper introduced and compared algorithms based on their respective accuracy and reproducibility. The estimation of flow rate using the DSA and/or fluoroscopic images is a clinically relevant problem; therefore, the identification of reliable algorithms and methods in this direction is of particular significance. The CC algorithm with GV curve fit had the best overall accuracy for time measurement. For optimal accuracy, DSA acquisition at 6 F/s needs to be used. This study has provided a necessary groundwork to conduct a small-scale clinical study for feasibility analysis based on the approaches and parameters outlined and may lead to the development of a novel imaging product for vascular access application.

## Figures and Tables

**Table 1:** Summarized key statistical parameters for PP and CC algorithms with/without curve fitting.

Imaging Mode, Sampling Rate	Algorithm	Without Curve Fit			With GV Curve Fit			*p* Value ^†^
		Bias [Lower Limit, Upper Limit] (mL/min)	95% CI |MQE| (%)	Corr.* (*r*)	Bias [Lower Limit, Upper Limit] (mL/min)	95% CI |MQE| (%)	Corr.* (*r*)	
DSA acquisition, 6 F/s	*PP*	−176 [−842, 490]	[29, 43]	0.574	−246 [−676, 184]	[21, 32]	0.773	0.175 ^‡^
	*CC*	−234 [−691, 223]	[25, 35]	0.731	−145 [−484, 195]	[18, 25]	0.865	0.017 ^§^
Fluoroscopic, 10 P/s	*PP*	−374 [−1098, 351]	[42, 67]	0.545	−339 [−891, 213]	[34, 51]	0.515	0.027 ^‡^
	*CC*	−205 [−930, 519]	[43, 59]	0.534	−198 [−722, 327]	[30, 44]	0.576	0.031 ^§^

* Pearson’s correlation coefficient (*r*) of calculated flow rate vs. in-line flow sensor measurement. ^†^ Statistical test of significance (Student’s *t* test) between algorithms (peak-to-peak vs. cross-correlation; ^‡^ without curve fitting, ^§^ with gamma-variate curve fitting, respectively).

## Data Availability

The data shown in this article are available upon request from the corresponding author.

## Supplementary Materials

The following are available online at https://www.mdpi.com/article/10.3390/diagnostics11101771/s1, Figure S1: Overall quantification error of PP algorithm with different curve-fitting models, Figure S2: Variation of quantification accuracy across different flow rates for PP algorithm with DSA acquisition, Figure S3: Bland-Altman plots for fluoroscopic imaging, Figure S4: Radiation burden for DSA acquisition and fluoroscopic imaging.

## References

[1] Bogunović H., Loncarić S. (2006). Blood Flow and Velocity Estimation Based on Vessel Transit Time by Combining 2D and 3D X-ray Angiography. Med. Image Comput. Comput. Assist. Interv..

[2] Bonnefous O., Pereira V.M., Ouared R., Brina O., Aerts H., Hermans R., van Nijnatten F., Stawiaski J., Ruijters D. (2012). Quantification of Arterial Flow Using Digital Subtraction Angiography. Med. Phys..

[3] Waechter I., Bredno J., Hermans R., Weese J., Barratt D.C., Hawkes D.J. (2008). Model-Based Blood Flow Quantification from Rotational Angiography. Med. Image Anal..

[4] Koirala N., Anvari E., McLennan G. (2016). Monitoring and Surveillance of Hemodialysis Access. Semin. Interv. Radiol..

[5] Whittier W.L. (2009). Surveillance of Hemodialysis Vascular Access. Semin. Interv. Radiol..

[6] Polkinghorne K. (2008). The CARI Guidelines. Vascular Access Surveillance. Nephrology.

[7] Fitts M.K., Pike D.B., Anderson K., Shiu Y.-T. (2014). Hemodynamic Shear Stress and Endothelial Dysfunction in Hemodialysis Access. Open Urol. Nephrol. J..

[8] Browne L.D., Bashar K., Griffin P., Kavanagh E.G., Walsh S.R., Walsh M.T. (2015). The Role of Shear Stress in Arteriovenous Fistula Maturation and Failure: A Systematic Review. PLoS ONE.

[9] Cheung A.K., Imrey P.B., Alpers C.E., Robbin M.L., Radeva M., Larive B., Shiu Y.-T., Allon M., Dember L.M., Greene T. (2017). Intimal Hyperplasia, Stenosis, and Arteriovenous Fistula Maturation Failure in the Hemodialysis Fistula Maturation Study. J. Am. Soc. Nephrol..

[10] Rothuizen T.C., Wong C., Quax P.H.A., van Zonneveld A.J., Rabelink T.J., Rotmans J.I. (2013). Arteriovenous Access Failure: More than Just Intimal Hyperplasia?. Nephrol. Dial. Transpl..

[11] Stolic R. (2013). Most Important Chronic Complications of Arteriovenous Fistulas for Hemodialysis. Med. Princ. Pr..

[12] MacRae J.M., Dipchand C., Oliver M., Moist L., Lok C., Clark E., Hiremath S., Kappel J., Kiaii M., Luscombe R. (2016). Arteriovenous Access Failure, Stenosis, and Thrombosis. Can. J. Kidney Health Dis..

[13] Bountouris I., Kritikou G., Degermetzoglou N., Avgerinos K.I. (2018). A Review of Percutaneous Transluminal Angioplasty in Hemodialysis Fistula. Int. J. Vasc. Med..

[14] Vascular Access 2006 Work Group (2006). Clinical Practice Guidelines for Vascular Access. Am. J. Kidney Dis..

[15] Forkert N.D., Fiehler J., Ries T., Illies T., Möller D., Handels H., Säring D. (2011). Reference-Based Linear Curve Fitting for Bolus Arrival Time Estimation in 4D MRA and MR Perfusion-Weighted Image Sequences. Magn. Reson. Med..

[16] Muir E.R., Watts L.T., Tiwari Y.V., Bresnen A., Shen Q., Duong T.Q. (2014). Quantitative Cerebral Blood Flow Measurements Using MRI. Methods Mol. Biol..

[17] Prevrhal S., Forsythe C.H., Harnish R.J., Saeed M., Yeh B.M. (2011). CT Angiographic Measurement of Vascular Blood Flow Velocity by Using Projection Data. Radiology.

[18] Holland C.K., Clancy M.J., Taylor K.J., Alderman J.L., Purushothaman K., McCauley T.R. (1996). Volumetric Flow Estimation In Vivo and In Vitro Using Pulsed-Doppler Ultrasound. Ultrasound Med. Biol..

[19] Strouthos C., Lampaskis M., Sboros V., McNeilly A., Averkiou M. (2010). Indicator Dilution Models for the Quantification of Microvascular Blood Flow with Bolus Administration of Ultrasound Contrast Agents. IEEE Trans. Ultrason Ferroelectr. Freq. Control.

[20] Grant E.G., Benson C.B., Moneta G.L., Alexandrov A.V., Baker J.D., Bluth E.I., Carroll B.A., Eliasziw M., Gocke J., Hertzberg B.S. (2003). Carotid Artery Stenosis: Gray-Scale and Doppler US Diagnosis—Society of Radiologists in Ultrasound Consensus Conference. Radiology.

[21] Oglat A.A., Matjafri M.Z., Suardi N., Oqlat M.A., Abdelrahman M.A., Oqlat A.A. (2018). A Review of Medical Doppler Ultrasonography of Blood Flow in General and Especially in Common Carotid Artery. J. Med. Ultrasound.

[22] Winkler P., Helmke K., Mahl M. (1990). Major Pitfalls in Doppler Investigations. Part II. Low Flow Velocities and Colour Doppler Applications. Pediatr. Radiol..

[23] O’Brien W.D.J. (2007). Ultrasound-Biophysics Mechanisms. Prog. Biophys. Mol. Biol..

[24] Ziskin M.C. (1993). Fundamental Physics of Ultrasound and Its Propagation in Tissue. Radiographics.

[25] Pinto A., Pinto F., Faggian A., Rubini G., Caranci F., Macarini L., Genovese E.A., Brunese L. (2013). Sources of Error in Emergency Ultrasonography. Crit. Ultrasound J..

[26] Stasi G., Ruoti E.M. (2015). A Critical Evaluation in the Delivery of the Ultrasound Practice: The Point of View of the Radiologist. Ital. J. Med..

[27] Piola M., Ruiter M., Vismara R., Mastrullo V., Agrifoglio M., Zanobini M., Pesce M., Soncini M., Fiore G.B. (2017). Full Mimicking of Coronary Hemodynamics for Ex-Vivo Stimulation of Human Saphenous Veins. Ann. Biomed. Eng..

[28] Bihari P., Shelke A., Nwe T.H., Mularczyk M., Nelson K., Schmandra T., Knez P., Schmitz-Rixen T. (2013). Strain Measurement of Abdominal Aortic Aneurysm with Real-Time 3D Ultrasound Speckle Tracking. Eur. J. Vasc. Endovasc. Surg..

[29] Polanczyk A., Klinger M., Nanobachvili J., Huk I., Neumayer C. (2018). Artificial Circulatory Model for Analysis of Human and Artificial Vessels. Appl. Sci..

[30] Polanczyk A., Podgorski M., Polanczyk M., Piechota-Polanczyk A., Stefanczyk L., Strzelecki M. (2019). A Novel Vision-Based System for Quantitative Analysis of Abdominal Aortic Aneurysm Deformation. Biomed. Eng. Online.

[31] Xaplanteris P., Fournier S., Keulards D.C.J., Adjedj J., Ciccarelli G., Milkas A., Pellicano M., van’t Veer M., Barbato E., Pijls N.H.J. (2018). Catheter-Based Measurements of Absolute Coronary Blood Flow and Microvascular Resistance: Feasibility, Safety, and Reproducibility in Humans. Circ. Cardiovasc. Interv..

[32] Khanmohammadi M., Engan K., Sæland C., Eftestøl T., Larsen A.I. (2019). Automatic Estimation of Coronary Blood Flow Velocity Step 1 for Developing a Tool to Diagnose Patients with Micro-Vascular Angina Pectoris. Front. Cardiovasc. Med..

[33] Zafar H., Sharif F., Leahy M.J. (2014). Measurement of the Blood Flow Rate and Velocity in Coronary Artery Stenosis Using Intracoronary Frequency Domain Optical Coherence Tomography: Validation against Fractional Flow Reserve. IJC Heart Vasc..

[34] Koirala N., Setser R.M., Bullen J., McLennan G. Blood Flow Measurement Using Digital Subtraction Angiography for Assessing Hemodialysis Access Function. Proceedings of the SPIE 10137, Medical Imaging 2017: Biomedical Applications in Molecular, Structural, and Functional Imaging.

[35] Koirala N., Setser R., Bullen J., McLennan G. (2017). Determination of Dialysis Access Patency Using 2D Angiographic Images. 2017 IEEE 17th International Conference on Bioinformatics and Bioengineering (BIBE), Washington, DC, USA, 23–25 October 2017.

[36] Blomley M.J., Coulden R., Bufkin C., Lipton M.J., Dawson P. (1993). Contrast Bolus Dynamic Computed Tomography for the Measurement of Solid Organ Perfusion. Investig. Radiol..

[37] Ionita C.N., Wang W., Bednarek D.R., Rudin S. (2010). Assessment of Contrast Flow Modification in Aneurysms Treated with Closed-Cell Self-Deploying Asymmetric Vascular Stents (SAVS). Proc. SPIE Int. Soc. Opt. Eng..

[38] Shpilfoygel S.D., Close R.A., Valentino D.J., Duckwiler G.R. (2000). X-ray Videodensitometric Methods for Blood Flow and Velocity Measurement: A Critical Review of Literature. Med. Phys..

[39] Argueta E.E., Paniagua D. (2019). Thermodilution Cardiac Output: A Concept over 250 Years in the Making. Cardiol. Rev..

[40] Zierler K. (2000). Indicator Dilution Methods for Measuring Blood Flow, Volume, and Other Properties of Biological Systems: A Brief History and Memoir. Ann. Biomed. Eng..

[41] Rosen L., Silverman N.R. (1973). Videodensitometric Measurements of Blood Flow Using Crosscorrelation Techniques. Radiology.

[42] Kalisz K., Buethe J., Saboo S.S., Abbara S., Halliburton S., Rajiah P. (2016). Artifacts at Cardiac CT: Physics and Solutions. Radiographics.

[43] Lieber B.B., Sadasivan C., Hao Q., Seong J., Cesar L. (2009). The Mixability of Angiographic Contrast with Arterial Blood. Med. Phys..

[44] Brands J., Vink H., Van Teeffelen J.W.G.E. (2011). Comparison of Four Mathematical Models to Analyze Indicator-Dilution Curves in the Coronary Circulation. Med. Biol. Eng. Comput..

[45] Thompson H.K.J., Starmer C.F., Whalen R.E., McIntosh H.D. (1964). Indicator Transit Time Considered as a Gamma Variate. Circ. Res..

[46] Bassingthwaighte J.B., Ackerman F.H., Wood E.H. (1966). Applications of the Lagged Normal Density Curve as a Model for Arterial Dilution Curves. Circ. Res..

[47] Stohanzlova P., Kolar R. (2017). Tissue Perfusion Modelling in Optical Coherence Tomography. Biomed. Eng. Online.

[48] Harabis V., Kolar R., Mezl M., Jirik R. (2013). Comparison and Evaluation of Indicator Dilution Models for Bolus of Ultrasound Contrast Agents. Physiol. Meas..

[49] Koirala N., McLennan G. (2017). Analysis of Noisy 2D Angiographic Images for Improved Blood Flow Rate Quantification in Dialysis Access. 2017 IEEE Western New York Image and Signal Processing Workshop (WNYISPW), Rochester, NY, USA, 17 Novvember 2017.

[50] Bland J.M., Altman D.G. (1986). Statistical Methods for Assessing Agreement between Two Methods of Clinical Measurement. Lancet.

[51] Chen M.-C., Tsai W.-L., Tsai I.-C., Chan S.-W., Liao W.-C., Lin P.-C., Yang S.J. (2010). Arteriovenous Fistula and Graft Evaluation in Hemodialysis Patients Using MDCT: A Primer. AJR Am. J. Roentgenol..

[52] Murphy E.A., Ross R.A., Jones R.G., Gandy S.J., Aristokleous N., Salsano M., Weir-McCall J.R., Matthew S., Houston J.G. (2017). Imaging in Vascular Access. Cardiovasc. Eng. Technol..

[53] Rt S.A., Brehmer K., Torkel B.B. (2020). Evaluation of Arteriovenous Fistula-Bilateral Computed Tomography Venography Combining Diluted and Undiluted Contrast Media Injection: A Case Study. Radiol. Case Rep..

[54] Huda W., Abrahams R.B. (2015). Radiographic Techniques, Contrast, and Noise in X-ray Imaging. AJR Am. J. Roentgenol..

[55] Crowhurst J., Whitby M. (2018). Lowering Fluoroscopy Pulse Rates to Reduce Radiation Dose during Cardiac Procedures. J. Med. Radiat. Sci..

[56] Shin J.H. (2020). Recent Radiation Reduction Strategies for Neurointerventionists. Neurointervention.

[57] Bratschitsch G., Leitner L., Stücklschweiger G., Guss H., Sadoghi P., Puchwein P., Leithner A., Radl R. (2019). Radiation Exposure of Patient and Operating Room Personnel by Fluoroscopy and Navigation during Spinal Surgery. Sci. Rep..

[58] Pereira V.M., Bonnefous O., Ouared R., Brina O., Stawiaski J., Aerts H., Ruijters D., Narata A.P., Bijlenga P., Schaller K. (2013). A DSA-Based Method Using Contrast-Motion Estimation for the Assessment of the Intra-Aneurysmal Flow Changes Induced by Flow-Diverter Stents. AJNR Am. J. Neuroradiol..

[59] Seifalian A.M., Hawkes D.J., Hardingham C.R., Colchester A.C., Reidy J.F. (1991). Validation of a Quantitative Radiographic Technique to Estimate Pulsatile Blood Flow Waveforms Using Digital Subtraction Angiographic Data. J. Biomed. Eng..

[60] Mulder G., Bogaerds A.C.B., Rongen P., van de Vosse F.N. (2011). The Influence of Contrast Agent Injection on Physiological Flow in the Circle of Willis. Med. Eng. Phys..

[61] Wu T.-H., Lin C.-J., Lin Y.-H., Guo W.-Y., Huang T.-C. (2013). Quantitative Analysis of Digital Subtraction Angiography Using Optical Flow Method on Occlusive Cerebrovascular Disease. Comput. Methods Programs Biomed..

[62] Pereira V.M., Ouared R., Brina O., Bonnefous O., Satwiaski J., Aerts H., Ruijters D., van Nijnatten F., Perren F., Bijlenga P. (2014). Quantification of Internal Carotid Artery Flow with Digital Subtraction Angiography: Validation of an Optical Flow Approach with Doppler Ultrasound. AJNR Am. J. Neuroradiol..

[63] Tenjin H., Asakura F., Nakahara Y., Matsumoto K., Matsuo T., Urano F., Ueda S. (1998). Evaluation of Intraaneurysmal Blood Velocity by Time-Density Curve Analysis and Digital Subtraction Angiography. AJNR Am. J. Neuroradiol..

[64] Brockow K. (2020). Reduced Iodinated Contrast Media Dose and Injection Speed for CT: How Much Does This Decrease the Risk of a Hypersensitivity Reactions?. Quant. Imaging Med. Surg..

[65] Fåhræus R., Lindqvist T. (1931). The Viscosity of the Blood in Narrow Capillary Tubes. Am. J. Physiol.-Leg. Content.

[66] Brindise M.C., Busse M.M., Vlachos P.P. (2018). Density and Viscosity Matched Newtonian and Non-Newtonian Blood-Analog Solutions with PDMS Refractive Index. Exp. Fluids.

[67] Huang T.-C., Chang C.-K., Liao C.-H., Ho Y.-J. (2013). Quantification of Blood Flow in Internal Cerebral Artery by Optical Flow Method on Digital Subtraction Angiography in Comparison with Time-of-Flight Magnetic Resonance Angiography. PLoS ONE.

[68] Cai J., Wu D., Mo Y., Wang A., Hu S., Ren L. (2016). Comparison of Extracranial Artery Stenosis and Cerebral Blood Flow, Assessed by Quantitative Magnetic Resonance, Using Digital Subtraction Angiography as the Reference Standard. Medicine.

[69] Wen W.-L., Fang Y.-B., Yang P.-F., Zhang Y.-W., Wu Y.-N., Shen H., Ge J.-J., Xu Y., Hong B., Huang Q.-H. (2016). Parametric Digital Subtraction Angiography Imaging for the Objective Grading of Collateral Flow in Acute Middle Cerebral Artery Occlusion. World Neurosurg..

