# Supplementary material for: Mathematical Models for Blood Flow Quantification in Dialysis Access Using Angiography: A Comparative Study
Source: Diagnostics (Basel). 2021 Sep 26;11(10):1771. doi: 10.3390/diagnostics11101771 (PMC8534972; doi:10.3390/diagnostics11101771)
Supplement: Supplementary file 1 [file diagnostics-11-01771-s001.zip › diagnostics-1362762-supplementary.pdf]

diagnostics

MDPI

Supplementary Materials

Citation: Koirala, N.; McLennan, G. Mathematical models for blood flow quantification in dialysis access using angiography: A comparative study. *Diagnostics* **2021**, *11*, 1771. https://doi.org/10.3390/diagnostics11101771

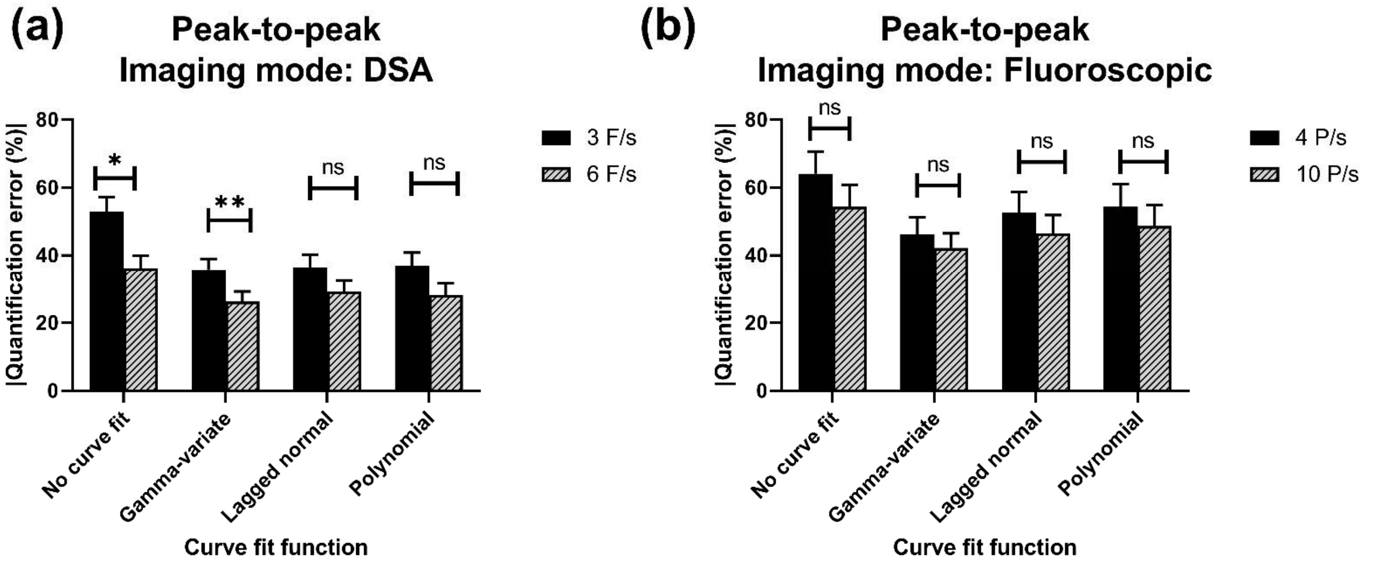

Figure S1. Overall quantification error of PP algorithm with different curve-fitting models. Quantification error with (**a**) DSA acquisition at 3 and 6 F/s, and (**b**) fluoroscopic imaging at 4 and 10 P/s. *–0.008, **–0.025, **ns**–non-significant.

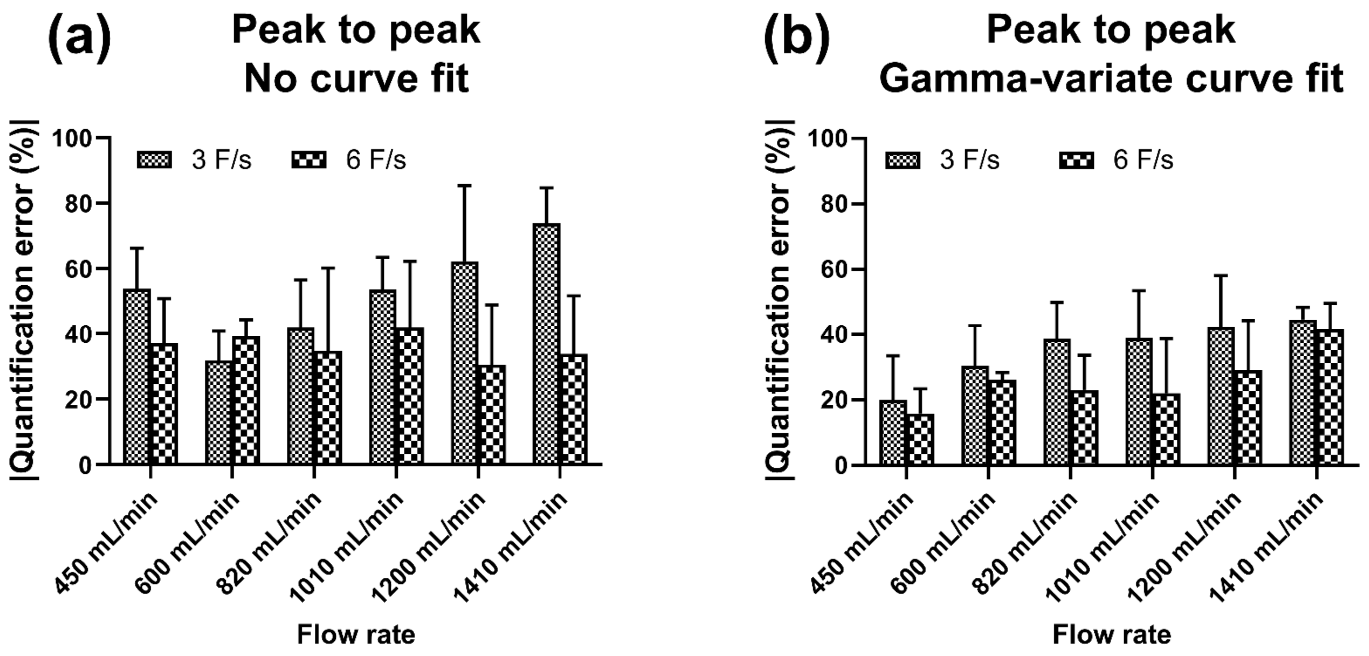

Figure S2. Evaluation of quantification accuracy of PP algorithm at different flow rates for DSA acquisition. Absolute quantification error (**a**) without curve-fit, and (**b**) with GV curve-fit.

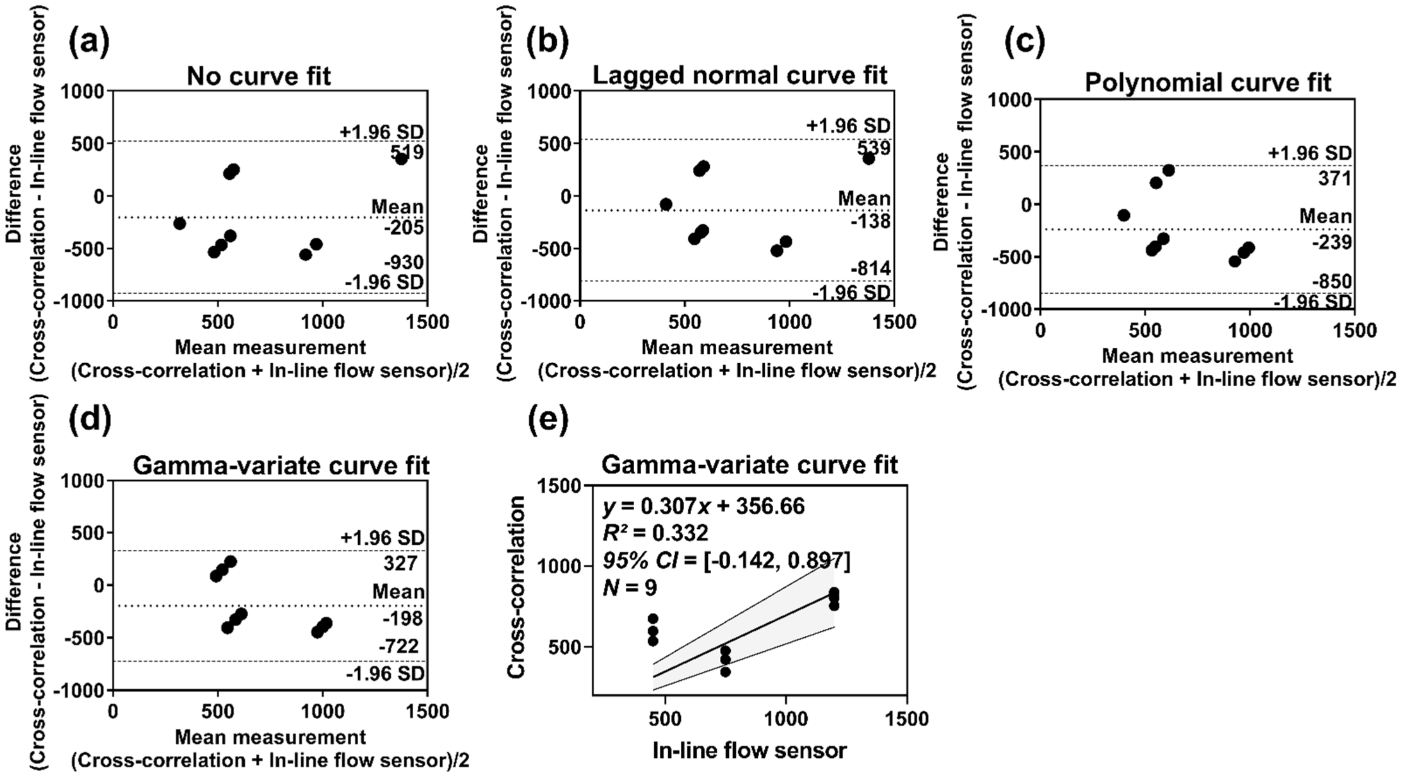

Figure S3. (**a**)−(**d**) Bland-Altman plots for fluoroscopic imaging showing the bias (mean) and limits of agreement (mean ± 1.96 × SD) of CC algorithm with/without curve-fitting versus in-line flow sensor measurement, (**e**) Scatter plot showing the correlation between measurements obtained with CC algorithm + gamma-variate curve-fit and in-line flow sensor, along with 95% confidence bands of the best-fit line.

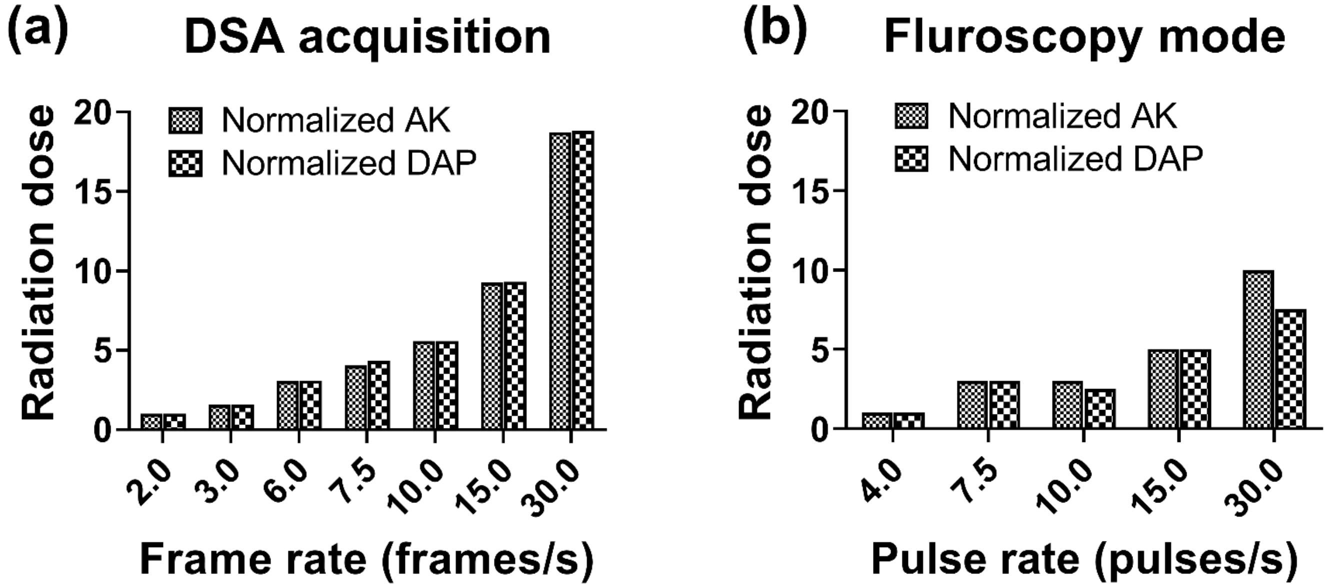

Figure S4. Radiation burden for (**a**) DSA acquisition, and (**b**) fluoroscopic imaging. The quantities were normalized with the respective radiation dose parameters (AK or DAP) at 2 F/s (DSA) and 4 P/s (Fluoroscopy). The actual dose at 2 F/s (DSA) was 0.158 mGy (AK) and 1.38 μGy.m² (DAP); the dose at 4 P/s (fluoroscopy) was 4 μGy (AK) and 0.04 μGy.m² (DAP). DSA, Digital subtraction angiography; AK, Air kerma; DAP, Dose area product (also called- Air kerma area product [AKAP]).
